# First Report of *Pseudobodo* sp, a New Pathogen for a Potential Energy-Producing Algae: *Chlorella vulgaris* Cultures

**DOI:** 10.1371/journal.pone.0089571

**Published:** 2014-03-05

**Authors:** Zhangran Chen, Xueqian Lei, Bangzhou Zhang, Luxi Yang, Huajun Zhang, Jingyan Zhang, Yi Li, Wei Zheng, Yun Tian, Jingwen Liu, Tianling Zheng

**Affiliations:** 1 State Key Laboratory for Marine Environmental Sciences and Key Laboratory of the Ministry of Education for Coastal and Wetland Ecosystem, School of Life Sciences, Xiamen University, Xiamen, China; 2 ShenZhen Research Institute of Xiamen University, ShenZhen, China; 3 Bioengineering College of Jimei University, Xiamen, China; Mount Allison University, Canada

## Abstract

*Chlorella vulgaris*, is a kind of single-celled green algae, which could serve as a potential source of food and energy because of its photosynthetic efficiency. In our study, a pathogenic organism targeting *C. vulgaris* was discovered. The algae-lytic activity relates to a fraction from lysates of infected *C. vulgaris* that was blocked upon filtration through a 3 µm filter. 18S rRNA gene sequence analysis revealed that it shared 99.0% homology with the protist *Pseudobodo tremulans*. Scanning electron microscope analysis showed that *Pseudobodo* sp. KD51 cells were approximately 4–5 µm long, biflagellate with an anterior collar around the anterior part of the cell in unstressed feeding cells. Besides the initial host, *Pseudobodo* sp. KD51 could also kill other algae, indicating its relatively wide predatory spectrum. Heat stability, pH and salinity tolerance experiments were conducted to understand their effects on its predatory activities, and the results showed that *Pseudobodo* sp. KD51 was heat-sensitive, and pH and salinity tolerant.

## Introduction

*Chlorella vulgaris*, a spherical single-celled freshwater alga, is widespread and is an efficient photosynthetic plant. It is highly favored for its high content of protein, vitamins, minerals, dietary fiber, nucleic acid, chlorophyll and other essential materials [1], [2]. Driven by the increasing price of petroleum-based fuels and growing concern about global warming, biofuels have aroused public attention [3]. Algal fuel or algal biofuel using algae as its source of natural deposits is an alternative to fossil fuel [4]. Hence, *C. vulgaris* not only plays an important role in aquatic ecology, but also has potential as an energy resource.

However, there are many factors that can affect the *in situ* growth of *C. vulgaris* [5], especially biotic factors [6], [7], [8], [9], which may result in a diminished output. In fact, the relationship between algae and other microorganisms including actinomycetes [10], [11], [12], viruses [13], [14] and bacteria [15] have been studied for decades. However, there was less literature about predatory-prey interactions between protists and algae. In many reports, harmful algae bloom regulation utilizing the special interactions revealed that protists may be a potential tool to control the harmful algae bloom [16], [17], [18], [19], [20]. However, unlike those red-tide species, *C.vulgaris* is a potentially valuable algae but there are no reports about the relationship between protists and *C. vulgaris*. We hope this study could provide a satisfactory basis to review the adverse factors for the growth of beneficial algae and then to look for methods to reduce these adverse effects to better serve for us.

The bicosoecids (Bicosoecea) are a small group of unicellular flagellates, included among the heterokonts in the kingdom *Chromista* [21]. The cells are free-living, with no chloroplasts, and in some genera are encased in a lorica. The Bicosoecida is an order of unicellular marine flagellates that have for the most part been poorly studied. Bicosoecids consume bacteria in the oceanic environment and may have a direct effect on deep-sea marine fluxes of nutrients [22]. While research continues on these organisms, much remains unknown about bicosoecids and other deep-water nanoflagellates [23]. Up to now, there are five genera in the family Bicosoecea; these are *Bicosoeca*, *Cafeteria*, *Pseudobodo*, *Siluania* and *Symbiomonas*. However, there is still little information about the last three genera. Some bicosoecid genera have been identified for decades, while some, especially the genus *Cafeteria*, have only recently been discovered. The genus was created after the discovery of a new species *Cafeteria roenbergensis*, a tiny (5–10 µm) eukaryotic organism that is eaten by protozoa and small invertebrates [24]. The name is meant to indicate the importance of the genus in the food web.

Another bicosoecid species is *Pseudobodo tremulans*, also a marine flagellate found mainly in sediments. At first, only loricate, sedentary flagellates of the genus *Bicosoeca* ( = *Bicoeca*) have been considered members of the group [22]. *P. tremulans* (Griessmann, 1913) Fenchel, 1982 is the first nonloricate flagellate to be referred to the bicosoecids [25]. The brief report of its ultrastructure contains few details, and few molecular sequences about *Pseudobodo* sp. data are available. Currently, there are no other reported species of *Pseudobodo* sp except *P.tremulans* and consequently, the relationships of this genus and other microorganisms are not well understood. The absence of ultrastructural and molecular data for many species belonging to, or thought to be related to, the bicosoecids has prevented a better understanding of bicosoecid taxonomy and phylogeny [26]. In this study, *Pseudobodo* sp., a new pathogen of *C. vulgaris*, was discovered and this study would be the first report concerning the relationship between the protist *Pseudobodo* and the eukaryotic alga *C. vulgaris*.

## Materials and Methods

### Algal cultures and growth conditions

The axenic clonal strain of *C. vulgaris* was cultured in f/2 medium (75 mg NaNO_3_, 5 mg NaH_2_PO_4_•H_2_O, 4.36 mg Na_2_EDTA•2H_2_O, 3.15 mg FeCl_3_•6H_2_O, 0.01 mg CoCl_2_•6H_2_O, 0.18 mg MnCl_2_•4H_2_O, 0.006 mg NaMoO_4_•2H_2_O, 0.1 mg Thiamine•HCl, 0.5 µg Vitamin B_12_, 0.5 µg Biotin, in 1 L seawater) [27] prepared with 0.45 µm of filtered seawater) at 20±1°C under a 12/12-h light-dark cycle of approximately 50 µmol of photons m^−2^s^−1^.

### Isolation of a predatory microbes

Water samples (0.5 m below the surface) in an algal bloom were collected from Xiamen sea of China on 1 August 2011. No specific permissions were required for these locations/activities, as Xiamen sea is a open sea area, not the private territory. We often collect samples from here when needed. The field studies related with the water sample collection did not involve endangered or protected species. The samples were immediately filtered through a 5-µm-pore-size filter, to remove large eukaryotic microorganisms. Then the treated samples were stored at 4°C for further analysis, which involved in 5 mL of the supernatant being inoculated into an exponentially growing *C. vulgaris* culture (25 mL) and incubated at 20°C using the same lighting conditions as mentioned above. Algal cultures inoculated with supernatant inactivated at 121°C served as controls. After *C. vulgaris* was inoculated with the water samples for about 14 days, the color of *C. vulgaris* had changed to be transparent and apparent aggregation of host formed at the bottom and the lysate of *C.vulgaris* had relatively stable alga-lytic activity. The responsible pathogen in the lysate was tested and purified through two extinction dilution cycles [28] and plaque assay procedure [29]. The dilution cycles were that, in brief, the algal lysate was diluted in modified f/2 medium in a series of 10-fold dilution steps. Aliquots (1 mL) of each dilution step were added to a test tube containing 4 mL of an exponentially growing *C.vulgaris*. Then, the algal lysate in the most dilution in the first assay was carried over to the second extinction dilution procedure. Lastly, the resultant lysate in the final endpoint dilution was used as a clonal lysate. As for plaque assay procedure, 4 mL of the upper medium (f/2+0.7% agar) with the addtion of 100 µL lysate and 1 mL *C.vulgaris*, were poured onto the lower medium (f/2+1.2% agar) which were adjusted to about 55°C before use. The formed single plaques were picked up and resuspended in f/2 overnight. Thereafter, 100 µL of suspension and 1 mL *C.vulgaris* together with 4 mL of the upper medium were again poured onto the lower medium. Pure pathogen were established by repeating the two methods several times.

### Pathogen size

To check whether the microbes in the lysate were viruses, bacteria or protists, and to determine the alga-lytic pattern, the lysate was filtered through 5, 3, 1, 0.45 and 0.2 µm-pore-size filter membrane. This is done to prepare for the determination of chlorophyll *a* content which could reflect the living mass. Then the supernatant (2 mL) was added into the exponentially growing host *C. vulgaris* (20 mL) while cultures inoculated with unfiltered lysate and f/2 served as the control. All tests had three replicates. After 7 days, all the cultures were centrifuged at 8,000×g at 4°C for 10 min. The supernatant was discarded, and the pellet was resuspended in 5 mL of 90% ethanol, mixed thoroughly on a vortex mixer and then rested overnight. After extraction, the pellet was removed by using centrifugation. The absorbance of the extracts was then measured at 664, 645, and 630 nm using a 50 Bio UV-Visible spectrophotometer (ELO71139140, Varian Australia Pty Ltd, Australia). Chlorophyll *a* concentration was calculated using the formula: Chlorophyll *a* (mgL^−1^) = 11.64A_664_-2.16A_645_-0.10A_630_ where A_664_, A_645_, and A_630_ are the optical densities at the respective wavelengths [30].

### DNA extraction

The pathogen was purified by using two extinction dilution cycles and the plaque assay procedure several times. For DNA extraction, the newly-prepared lysate of *C.vulgaris* that contained *Pseudobodo* sp. KD51 (30 mL) were centrifuged at 8,000× g at 4°C for 10 min and the supernate was discarded. The precipitate of the subsamples were mixed with 0.5 mL DNA buffer and incubated with 50 µL at 37°C for 1.5 h (50 mM Tris, 20 mM EDTA, 100 mM Na_3_PO_4_•H_2_O_,_ 1.5 M NaCl, 2% CTAB). SDS (10%) was added into the mixture and it was incubated at 65°C for 2.5 h with shaking every 15 min. Then 0.7 M NaCl and 1% CTAB/NaCl were added and the mixture further incubated at 65°C for 15 min. Isometric phenol-chloroform-isoamyl alcohol (25∶24∶1) was added and the phases were mixed gently and then separated by using centrifugation at 12,000× g for 10 min. This step was repeated twice. The aqueous phase was precipitated with isometric chloroform-isoamyl alcohol (24∶1) and collected by using concentrifugation at 12,000× g for 10 min. Afterwards, the mixture was precipitated by 0.5 volume isopropanol at 4°C for at least 1 h. Finally, the pellet was washed with 70% ethanol, dried and dissolved in 10 mM Tris pH 8.0, 1 mM EDTA and 0.1 µg µL^−1^. The purity of the extracted DNA was assessed spectrophotometrically by calculating the A_260_/A_280_ ratio to determine protein impurities. [31] The DNA was loaded on a 1.0% agarose gel to determine size and concentration.

### Polymerase chain reaction (PCR) and phylogeny construction

PCR was carried out in a 25 µL reaction volume containing 12.5 µL of Dream Taq Green PCR Master Mix (Thermo Scientific) and 0.8 µM of each primer from the relevant primer pair. SSU rDNA was amplified using the protozoa-specific primer P-SSU-342f (5′-CTTTCGATGGTAGTGTATTGGACTAC-3′) and the universal eukaryotic primer Medlin-B (5′-TGATCCTTCTGCAGGTTCACCTAC-3′ [32]) as used by Tan et al., (2013) [33], which yielded an approximately 1.5 kb portion of the SSU rDNA. The PCR condition for SSU were initial denaturation for 2 min at 94°C, 35 cycles of 45 s at 94°C, 45 s at 50°C, and 90 s at 72°C, and then terminal elongation for 5 min at 72°C. The PCR products were then loaded to 1.5% agarose gel to verify success of the PCR. Purification of the PCR product was carried out following the protocol of the TIANquick Midi purification kit (TIANGEN, China). Sequences of related taxa were downloaded from the GenBank database and the EzTaxon-e server (http://eztaxon-e.ezbiocloud.net/) [34]. Phylogenetic analysis was performed using MEGA version 5 [35] after multiple alignment of data with DNAMAN (version 5.1). Evolutionary distances and clustering were constructed by using the neighbor-joining method [36]. The resulting tree topology was evaluated using bootstrap analysis based on 1000 replicates.

### Impact and predatory activity of lysate on *C.vulgaris* cultures

In order to choose a proper volume ratio for further research, different volume ratios of newly-prepared lysate-*C.vulgaris* cultures (1∶1, 1∶10, 1∶20, 1∶50, 1∶100, 1∶500) were co-cultured in the 20 mL triangular flask while the *C. vulgaris* culture inoculated with f/2 under same ratio served as the control. After 9 days, all the cultures were treated as described above to measure the Chlorophyll *a* concentration. Next, the proper volume of the lysate was added into the exponentially growing host and the Chlorophyll *a* concentration wasn sequentially measured every day for 9 days to test the predatory activity of lysate on *C. vulgaris* cultures.

### Electron microscopy

In brief, the lysate were added into the exponentially growing host culture, and a fresh host culture inoculated in f/2 served as the control. An aliquot of cell suspension was sampled on day 5 when apparent infection appeared. Cells were then harvested by using centrifugation at 8,000×g at 4°C for 10 min and fixed with 1% glutaraldehyde for 4 h at 4°C and then washed in 0.1 M phosphate buffer (pH = 7.2), dehydrated through a graded ethanol series (30, 50, 70, 90, 95 and 100%), and embedded in Quetol 812 resin. Scanning electron microscope (SEM) images were taken on a Hitachi S-4800 microscope, operating at 5 kV.

### Predation range of *Pseudobodo* sp. KD51

25 exponentially growing clonal algal species were included to test the host specificity of *Pseudobodo* sp. KD51 by adding 5% (vol/vol) aliquots of fresh lysate. They were cultured under the conditions mentioned above at 20°C for 14 days. The growth condition of some of the algal species treated with *Pseudobodo* sp. KD51 were visually observed based on the color change, while other species were monitored by measuring the fluorescence value under a spectrophotometer or observed using optical microscopy.

### The standard curve of *C.vulgaris* numbers

Since the small size of *C. vulgaris* makes numerical counting under optical microscopy unsuitable, we searched for a convenient and direct way to monitor the change in numbers of *C. vulgaris*. In our study, we tried to build a standard curve of the numbers of live *C. vulgaris* cells by integrating the method of fluorescence microscope with SYBGreen as fluorescence stain and fluorescence value determination using by a spectrophotometer (Ex = 440 nm, Em = 680 nm). In brief, the initial maximum concentration of *C. vulgaris* (fluorescence value = 1122, regarded as “1”) was diluted in the proportions 3/4, 1/2, 3/8, 1/4, 3/16 and 1/8, and then the numbers of live cells in 200 µL of each dilution were assessed by counting, using the described two methods. The datas were analysed further and we found a significant correlation between the two sets. Therefore, we decided to assess the cell numbers directly by measuring the fluorescence value.

### Stability of predatory activity

To investigate the effect of heat treatment on predatory activity, the lysate was incubated in a water bath at 20, 25, 30, 35, 40, 45, 50 and 60 for 30 min, while another lysate was autoclaved at 121°C for 30 min. In addition, in order to investigate the effect of pH, the supernatants (initial pH = 7.8) were suspended in 0.1 M citrate phosphate buffer in a pH range of 5.5 to 9.5 for 30 min and then readjusted to the initial pH. The treated supernatants were subsequently inoculated into exponentially growing culture of *C.vulgaris*, to measure their fluorescence value. *C.vulgaris* was inoculated with *Pseudobodo* sp. KD51 salinities (0, 10, 20, 30, 40, 50 and 60‰) to test whether salinities could affect the activity of *Pseudobodo* sp. KD51. Subsamples of 200 µL were collected to determine the fluorescence value.

## Results

### Isolation of the predatory pathogen

Filtered seawater (5 mL) was added to the exponentially growing axenic clonal strain *C. vulgaris* cultures (20 mL) and the same volume of inactivated supernatant served as the control. It was observed that the color of the algal culture changed from green to light yellow, with algae being precipitated at the bottom after 5 days. In order to eliminate any accidental chance, the newly infected lysate was added into the host again. Consequently, the same algal death process was observed (Fig. S1A). Furthermore, plaque-forming assay of the lysate was performed on algal plates, and all plates developed plaques with a range of 2 to 3 mm in diameter (Fig. S1B). By repeating this procedure several times, we believed that we had isolated a pure plaque-forming microbe. We subsequently focused our attention on its biological characteristics.

### Size of the pathogen and its alga-lytic mode

**Figure 1:**
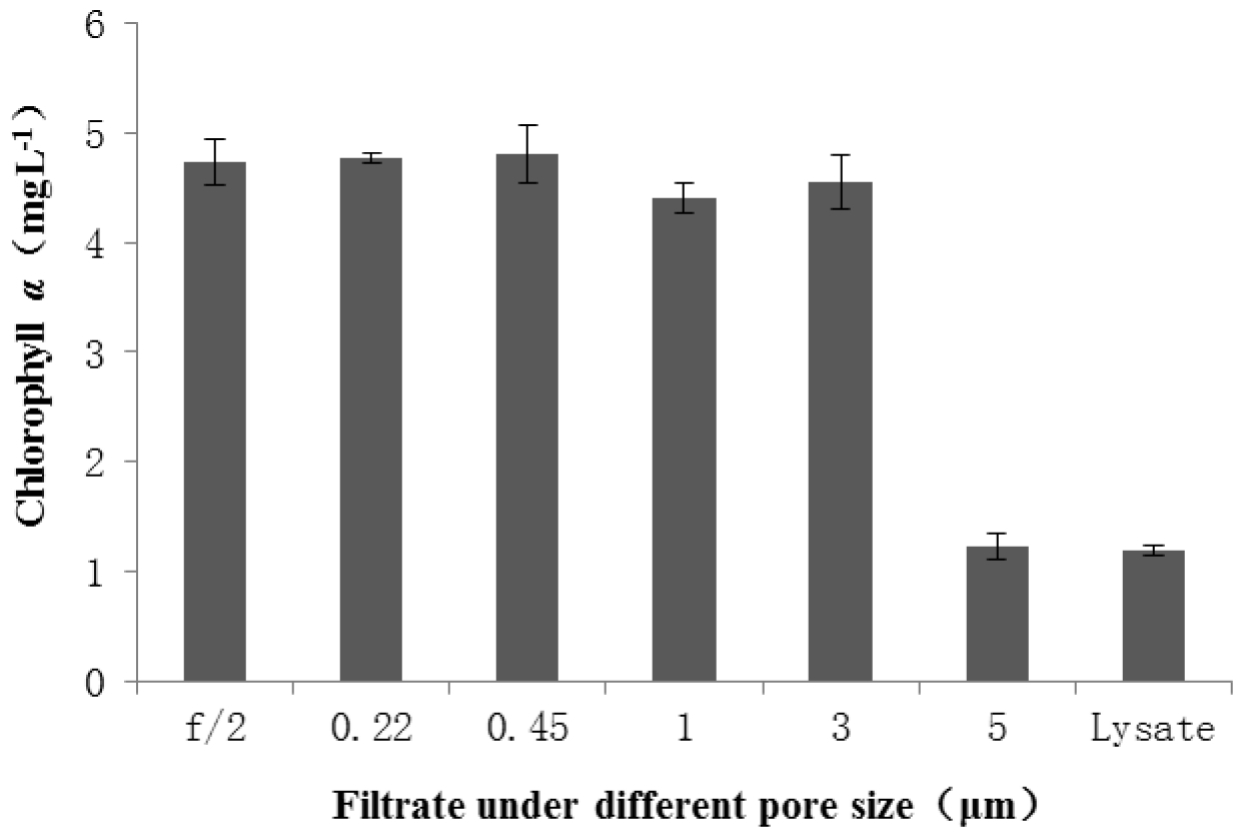
Size of the pathogen inferred from the results of predatory effects of lysate of *C. vulgaris* on *C. vulgaris*. The different pore-sized membranes, 0.22 µm, 0.45 µm, 1 µm, 3 µm, 5 µm were used and the legend f/2 meaning straight media with no *Pseudobodo* sp. KD51 served as the control. *C. vulgaris* was inoculated with both the control and treatment group for about 8 days, thereafter, the Chlorophyll *α* was determined. All data were mean± 1.SE (n = 3).

The lysate was filtered through different pore-sized membranes and the filtrate was added into the exponentially growing *C.vulgaris*, while f/2, the straight media with no pathogen served as control. Our results using the filtrates from different-pore sized filter membranes to infect the *C. vulgaris* revealed that the size of the pathogen was between 3–5 µm, as shown in Fig. 1. Compared with the control group, there was little change in the content of Chlorophyll *a* among the membrane pore sizes 0.22–3 µm, which meant that *C. vulgaris* could not be infected by the filtrate through a membrane less than 3 µm. However, both the untreated and 5 µm-sizes filtered samples showed similar predatory effects, which could be reflected in the largely decreased Chlorophyll *a* content.

### The predatory effect of lysate on *C. vulgaris* cultures

**Figure 2:**
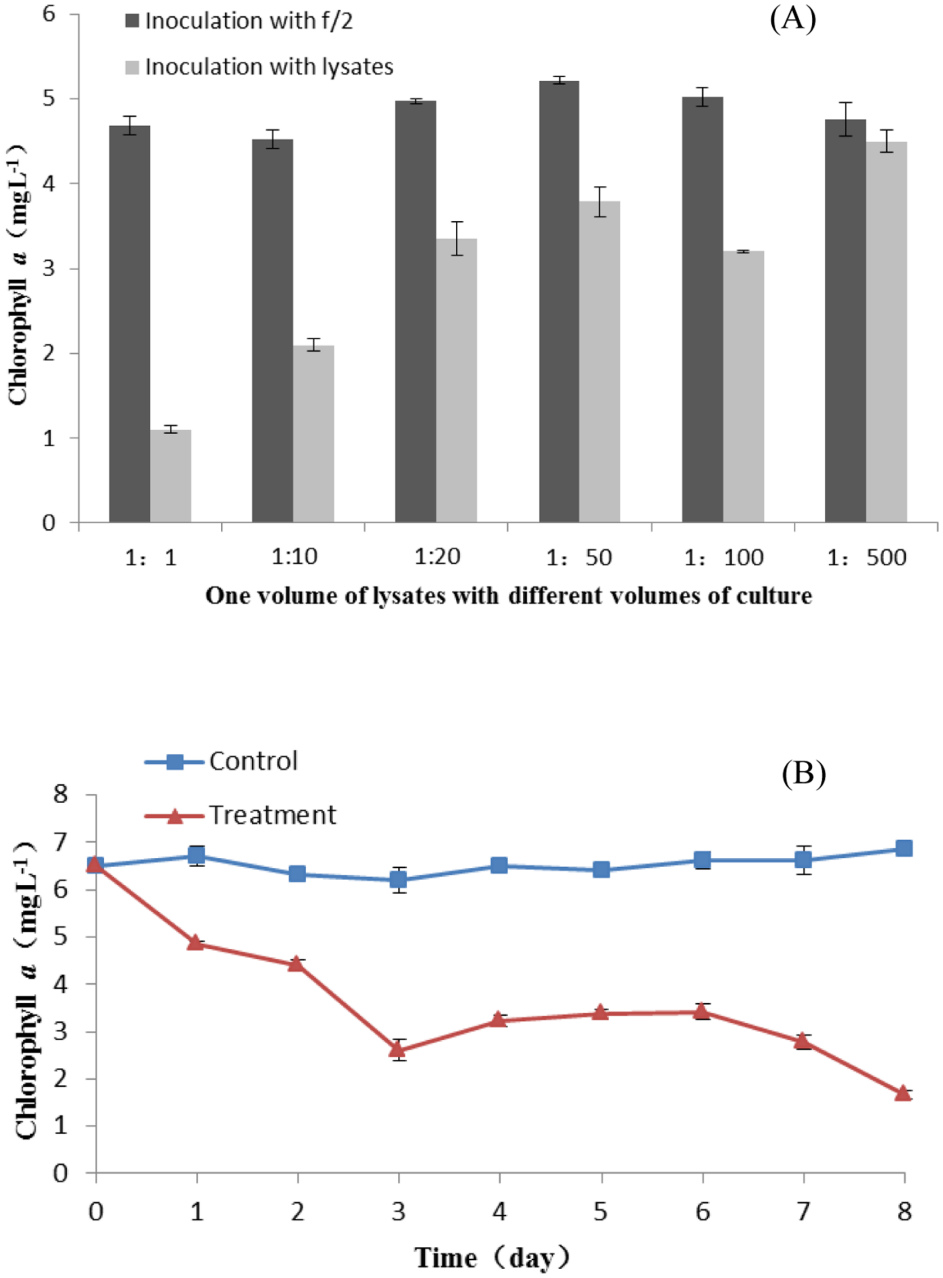
The predatory effect of lysate of *C. vulgaris* on *C. vulgaris* cultures. The cultures inoculated with f/2 served as control. All data were mean±1. SE (n = 3). (A). Inoculation of lysate of *C.vulgaris* with *C. vulgaris* under different volume ratio for about a week. (B). Inoculation of lysate with *C. vulgaris* under the ratio 1∶10 and the Chl *a* content was measured before addition of lysate and for 8 days thereafter.

The samples inoculated with different concentrations of lysate all showed different predatory level and the activities were higher as the concentration of lysate was higher (Fig. 2A). As can be seen, after 8 days of infection, the Chlorophyll *a* content of the treatment 1∶1 sharply decreased while in fact the vivid death was observed in the 2nd day (Fig. S2). The Chlorophyll *a* content of 1∶10 decreased largely and the vivid death was observed in the 4th day. There was slightly reduction of Chlorophyll *a* content for 1∶500 in the last day and there was also no vivid change compared with the control. We then chose the moderate volume ratio (1∶10) for the following study. In order to understand how *C. vulgaris* reacted to the pathogen, the Chl *a* content was measured before addition of lysate and for 8 days thereafter. The cultures inoculated with f/2 served as control. In the control, there were almost no vivid changes in the Chlorophyll *a* content. However, after the inoculation with the lysate, the Chlorophyll *a* content gradually decreased from 6.5 mg/L (0) to 2.5 mg/L (3rd) and there was slight fluctuation during 3rd and 7th, then decreasing to 1 mg/L at last.

### Molecular data concerning the pathogen

**Figure 3:**
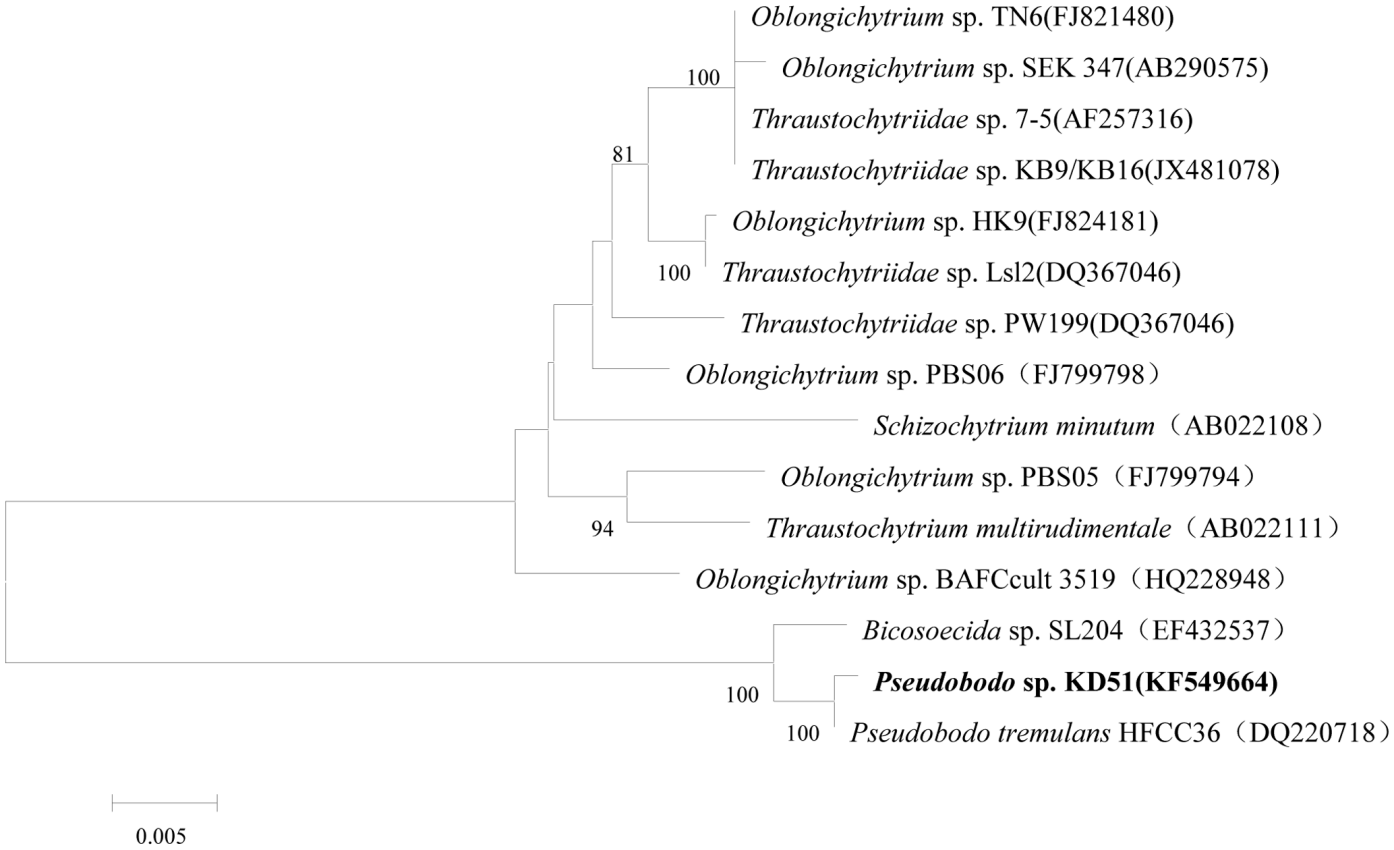
Phylogenetic tree based on the 18S rRNA gene sequence showing the relationship between *Pseudobodo* sp. KD51 and other sequences. The tree was constructed using the neighbor-joining method and the MEGA 5.0 program. Bootstrap values above 70% (expressed as percentages of 1000 replications) are given at nodes. The bootstrap values were evaluated from 1,000 replications.

SSU rRNA gene sequences from a wide representation of stramenopile lineages, were analyzed to assess the phylogenetic relationship of *Pseudobodo* sp. KD51 within this assemblage. Preliminary comparison of the 18S rRNA gene sequence of (1496 bp) *Pseudobodo* sp. KD51 (KF549664) with other sequences indicated that it was most closely related to the *P. tremulans* (99%). Sequence similarity between *Pseudobodo* sp. KD51 and other members of the *Bicosoecida* were slightly lower (98%). However, distant similarity (≤89%) was seen between *Pseudobodo* sp. KD51 and members from the *Labyrinthulomycetes*, such as *Oblongichytrium* sp. and *Thraustochytriidae* sp. The related sequences were chosen for phylogenetic tree construction. As shown in Fig. 3, *Pseudobodo* sp. KD51 only clustered within the *Bicosoecida* group branch, while other branches mainly consisted of species from the *Labyrinthulomycetes*.

### Morphology

**Figure 4:**
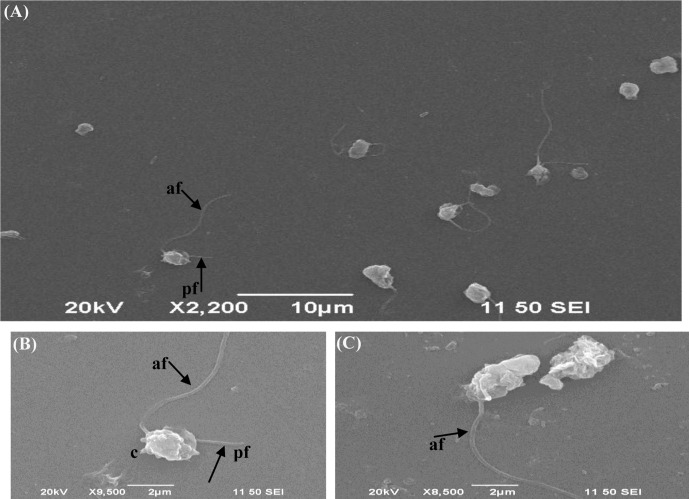
SEM view of *Pseudobodo* sp. KD51 under different fields. There were two unequal flagella around the feeding cells. The insertion sites of the two flagellae (af and pf) were separated by a protrusion at the anterior of the cell (c). (A) The figure of *Pseudobodo* sp. KD51 within the lysate of *C.vulgaris* under lower magnification. (B) and (C) were the magnified image of part in (A), the pf was missing (C) as the reason of sample preparation.The abbreviations: af–anterior flagellum, pf–posterior flagellum, c– cell. Magnification: (A): 2,200×; (B): 9,500×; (C): 8,500×.

From Fig. 4A, we saw that the *Pseudobodo* sp. KD51 cells were about 4–5 µm long with an anterior collar around the anterior part of the cell in unstressed feeding cells. The insertion sites of the two flagella were separated by a protrusion at the anterior of the cell (Fig. 4B). The anterior flagellum had a sine-wave beating pattern and was about 3 times the length of the cell, while the posterior flagellum was about twice the length of the cell and became attached to the substrate at its tip. The cells moved by swimming with the anterior flagellum directed forwards (Fig. 4C).

### Predatory range of *Pseudobodo* sp. KD51

When the host range of *Pseudobodo* sp. KD51 was tested using 25 algal species, predatory activity was apparent not only to the host alga *C. vulgaris*, but also to four other alga, *Platymonas subcordiformis*, *Dunaliella salina* and *Cyanobacteria* sp *Microcystis aeruginosa* (Table 1). Weak predation activity to *Isochrysis galbana and Nannochloropsis oceanica* was also detected through calculating the predatory rate. These results indicated the relatively wide predatory spectrum of *Pseudobodo* sp. KD51.

**Table 1:** Effects of *Pseudobodo* sp. KD51 on other microalgae.

Target species		Predatory activity	
		KD51	Control
Chlorophyta	*Dunaliella salina*	+	―
	*Platymonas subcordiformis*	**+**	―
	*Chlorella vulgaris*	***+***	―
	*Platymonas helgolandica*	―	―
	*Prasinophyceae*	―	―
	*Chlorella*	―	―
Bacillariophyta	*Phaeodactylum tricornutum*	―	―
	*Chaetoceros compressus*	―	―
	*Thalassiosira pseudonana*	―	―
	*Pmphiprora alata*	―	―
	*Thalassiosira weissflogii*	―	―
	*Asterionella japonica*	―	―
	*Skeletonema tropicum*	―	―
Pyrrophyta	*Alexandrium catenella DH01*	―	―
	*Alexandrium tamarense DH01*	―	―
	*Alexandrium minutum TW01*	―	―
	*Scrippsiella trochoidea XM01*	―	―
	*Prorocentrum donghaiense*	―	―
Cyanobacteria	*Microcystis aeruginosa*	+	―
Xanthophyta	*Heterosigma akashiwo*	―	―
	*Chattonella marina*	―	―
Chrysophyta	*Phaeocystis globosa*	―	―
	*Dicrateria inornata*	―	―
	*Isochrysis galbana*	W	―
	*Nannochloropsis oceanica*	W	―

—: Negative predation activity; +: Positive predation activity; W: Weakly positive predation activity.

### The standard curve of *C.vulgaris* numbers

**Figure 5:**
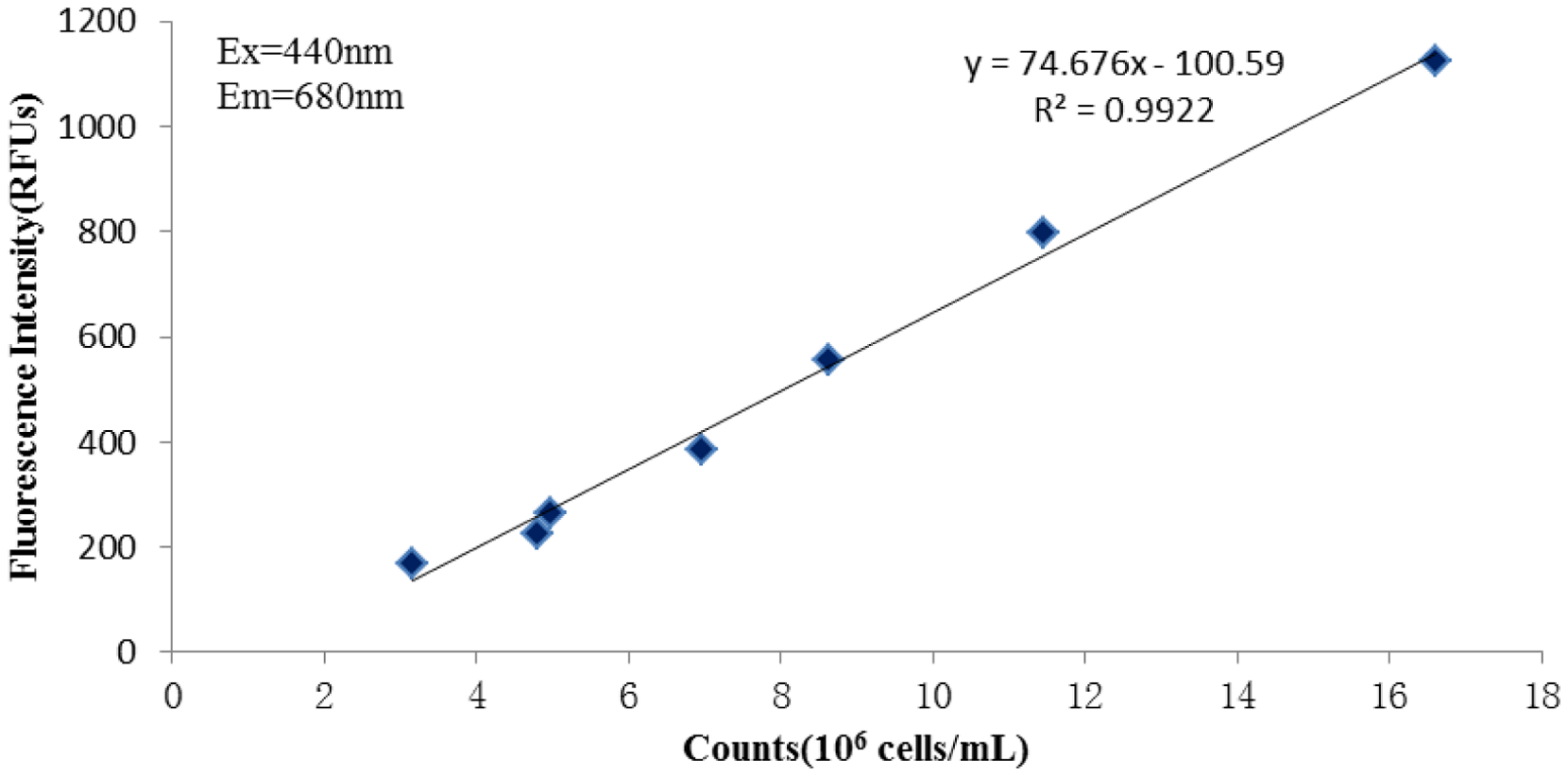
Standard curve of numbers of *C. vulgaris*. The initial *C.vulgaris* cells were diluted to different proportion 3/4, 1/2, 3/8, 1/4, 3/16, 1/8 separately by sterilized distilled water. Then the each diluted sample was partly counted by Fluorescence microscope with SYBGreen as fluorescence stain (X axis). Another part were determined by Spectrophotometer under the Ex (Excitation wavelength) = 440 nm and Em (Emission wavelength) = 680 nm, the determined value, Fluorescence intensity (Y axis) could indirectly reflect the numbers of the cells.

The two sets of data regarding cell numbers were collected and further analyzed. The standard curve was credible (Fig. 5) as it showed a significant correlation (R^2^ = 0.9922). In brief, the X-axis reflected the numbers of *C. vulgaris* cells counted using fluorescence microscopy, while the Y-axis reflected the fluorescence value by a spectrophotometer under the conditions as described above. Because the details of sample preparation for fluorescence microscopy were relatively complicated, in the following study we directly measured the fluorescence value using a spectrophotometer.

### Stability of predatory activity

**Figure 6:**
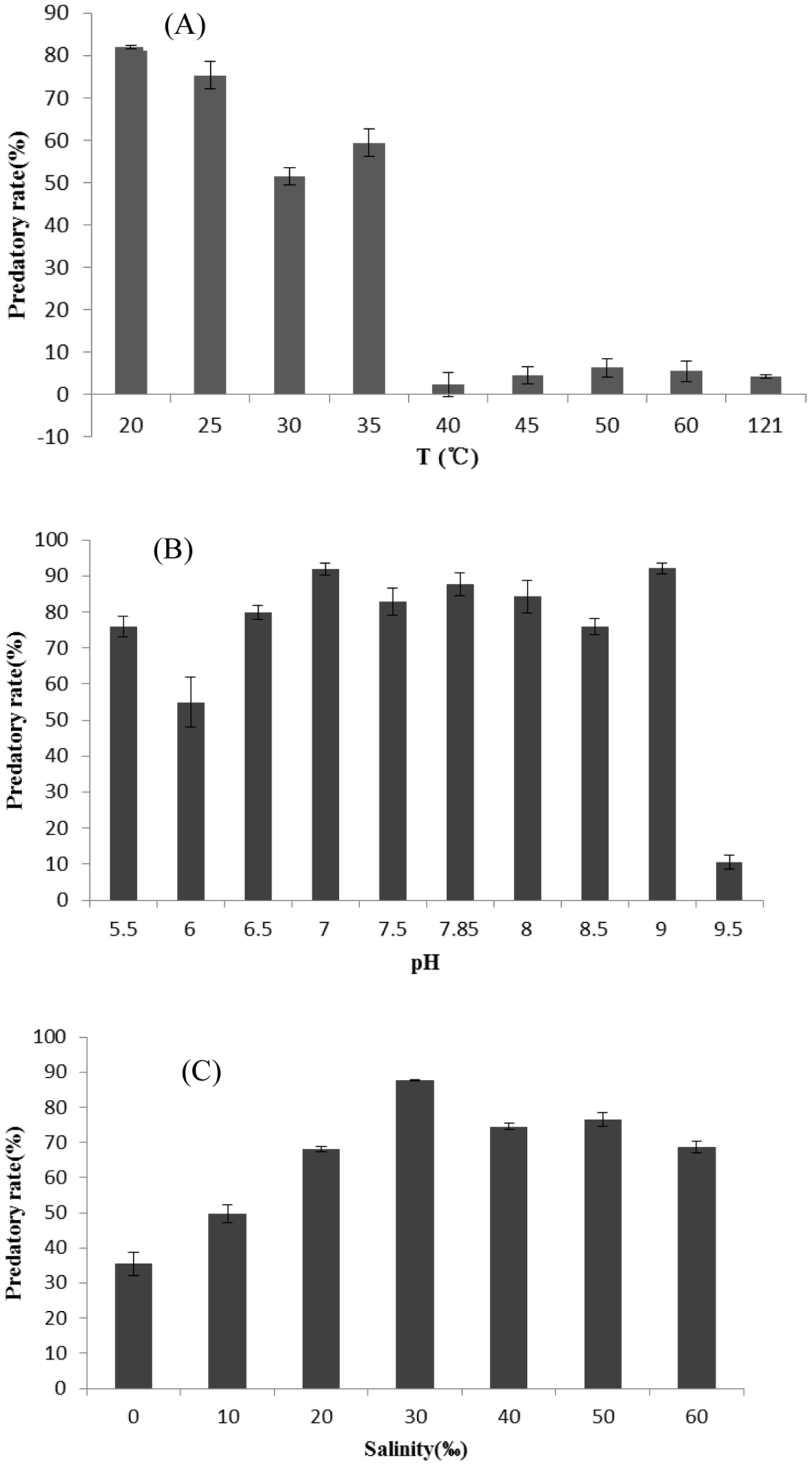
Stability of predation activity of the lysate of *C. vulgaris* under different physicochemical conditions. (A). Temperature from 20–121°C. For temperature stability, lysate of *C. vulgaris* were inoculated and kept at different temperatures for 1 h and then the treated samples were added into the *C. vulgaris* cultures. (B). For pH stability, pH of lysate (initial pH = 7.85) were adjusted to other pH values (5.5–9.5). After being kept for 1 h, the pH of each was readjusted to pH = 7.85. (C). For salinity stability, the salinity of the lysate (initial salinity = 30‰) were adjusted to other salinity values and kept for 1 h, then readjusted to the initial salinity.

The stability of predatory activity following heat treatment was tested and lysate after incubation at a higher temperature (>40°C) for 30 min did not exhibit predatory activity (Fig. 6A), indicating that *Pseudobodo* sp. KD51 was heat-sensitive. Lysate treated with pH between 5.5 and 8.0 (Fig. 6B) maintained a relatively high predatory activity but the activity decreased sharply at higher pH (9.5), suggesting that *Pseudobodo* sp. KD51 was relatively tolerant to pH. The rate increased along with the increasing salinity gradient (from 0 to 30‰) while the predatory rate fluctuated at 80% between 40 and 50‰ salinity (Fig. 6C).

## Discussion

In early August 2011, an algal bloom, caused mainly by *Heterosigma akashiwo* and *Skeletonema costatum* broke out in the Xiamen sea, China. Water samples were collected between the generation and termination of the bloom and pretreated for the isolation of alga-inhibiting microorganisms. After several cycles of purification using plaque assay procedure, plaques with a diameter of 2 mm on the *C.vulgaris* cultures led us to believe that we had obtained a stable alga-pathogen system. There are few reports in the literature concerning the effects of inhibitory microorganisms on *C. vulgaris*, and most of them describe the relationship between the algae and bacteria [7], [37], with little related to viruses [38], [39], [40], [41], and no literature describing the relationship between a protist and *C. vulgaris*. Our study confirmed that the pathogen we isolated could cause the etiolation and aggregation of *C. vulgaris* at the bottom of the flask. To further understand the biological characteristics of this pathogen (*Pseudobodo* sp. KD51) and its relationship with the alga, the molecular features and the morphology (using SEM) were determined, as well as the predatory spectrum and potential. Furthermore, its stability by the treatment of different abiotic factors were tested. To our knowledge, this is the first report of this pathogen's predatory capability against *C. vulgaris*.

Until now, most studies have focused on inhibitory activities against toxic algal species, while studies focusing on the microorganisms against energy microalgae are few. Their findings indicated that the effects of bacteria, viruses and protists on alga might be species-specific [12], [42]. In our study, among the 25 host alga for the predatory range test, three alga from *Chlorophyta* were susceptible to *Pseudobodo* sp. KD51 which implied that it had a relative broad predatory spectrum and might bring potential harm to the aquatic ecosystem through exerting influence on the important primary production. It is reported that predation in aquatic microbial food webs is dominated by phagotrophic protists, yet these microorganisms which exhibit a wide array of feeding strategies are still understudied compared to bacteria and phytoplankton. In addition, protistan grazing on bacteria is an important mechanism of nutrient regeneration, in particular, of nitrogen and phosphorus which arouse great attention about the relationship between protists and bacteria [43]. Research about the effects of protist on alga are still rare and the reason for the relatively wide alga-predatory spectrum of *Pseudobodo* sp. KD51 are complexed and awaits for further study.

The results using different sized membrane filters showed that the size of the pathogen was 3–5 µm which excluded the possibility of the pathogen being a virus, since the biggest virus observed previously involved the approximately 1 µm, pandoravirus-like particles [44], [45]. Also, it killed the alga through a direct way mechanism, since the lysate had no predatory activity after filtering through the 0.22 µm membrane. However, there are few reports concerning the direct inhibitory mechanism, and most of research is about the indirect inhibitory mechanism [46], [47], [48]. As protistan trophic states run the gamut from strictly phagotrophic, to mixotrophic: partly autotrophic and partly phagotrophic, to primarily autotrophic but capable of phagotrophy [49], but which way for *Pseudobodo* sp. KD51 is not sure. Furthermore, protists often feed directly on bacterial primary producers from the autotrophic picoplankton, that is, on free-living unicellular cyanobacteria that are mainly affiliated to the genera *Synechococcus* and *Prochlorococcus*, and on small eukaryotic alga such as *C. vulgaris* in this study [50]. The predatory mechanism and efficiency of protists are varied depending on the sizes of prey [51], [52], [53]. Our molecular results showed that the pathogen was more related with *P. tremulans* with a size between 3–5 µm, the size of which is almost the same as its prey *Chlorella vulgaris*. Hence, whether predatory mechanism of the pathogen is strictly or partly phagotrophic and mixotrophic also needs further study to confirm. However, we had reasons to speculate that it was the pathogenic protist that attacked the alga based on the following reasons.

In fact, we had not thought of the protist to be the pathogen at first, as our lab has always investigated the relationship between algicidal bacteria and algae [54], [55]. However, no gene fragment was amplified by the primer both from bacteria-specific (data not shown). Universal bacterial primers 27f/1492r [56] were used to exclude the possibility of the pathogen to be bacteria. After excluding the virus and bacteria, we suspected the pathogen may be a protist. SSU rDNA sequences based on a protozoa-specific primer could only be obtained from the DNA samples within the lysate while no band was available from the normal growing *C.vulgaris* without pathogen, suggesting that this primer pair had a high credibility. The primer amplifying the fragment revealed that the pathogen *Pseudobodo* sp. KD51 was most closely related to *P. tremulans* (99%). The flagellate *P. tremulans* was described by Griessmann (1914) from various marine enrichment cultures and refound by Ruinen (1938). Later, a form isolated from Aarhus Bay and from the Limfjord, where it is a numerous and constant part of the plankton, was in all probability identical to Griessmann's organism based on the original description [25]. As reported by Sherr, predation in aquatic microbial food webs was dominated by phagotrophic protists [49]. Several reports about the role of protist in red-tide had revealed the importance of protist as a powerful tool [57], [58]. However, there were almost no reports directly describing the protists and energy alga. Green alga, have long been recognized as potentially good sources for biofuel production because of their high oil content and rapid biomass production. The average fatty acid contents of the algal oils are 36% oleic (18∶1), 15% palmitic (16∶0), 11% stearic (18∶0), 8.4% iso-17∶0, and 7.4% linoleic (18∶2) [59], [60], [61]. As the energy problem becomes more and more urgent, measures should be taken to remove the adverse factors for the growth of energy alga or enhance the resistant ability of alga to other negative parts using genetic transformation method [62], [63].

Other evidence confirming the pathogen to be the bicosoecid *Pseudobodo* was the morphological features. The flagellate was described by Griessmann, and accurate morphological features were reported. As stated by Fenchel (1982), the flagellate is some 4 to 5 µm in diameter and the trophic cells are egg or pear-shaped, with two flagella. A posterior, smooth one passes through a ventral furrow and is used for temporary attachment to particles or to the water film. In addition, a systemic description based on light and electron microscopy was conducted [22], which reveals that the trophic cells of *P. tremulans* are pear or egg-shaped, with a broad posterior end; two flagellae of unequal length emerge from the base of a small apical papilla or “collar”; cells are 5–8 µm long; the anterior flagellar length is 12–15 µm and the posterior flagellar length is 8–10 µm. All these studies describe in detail the general ultrastructure of *P. tremulans*. Fenchel (1982) investigated a few ultrastructural characteristics of *P. tremulans*, which basically parallel those described in our study. Both strains have an identical, bifurcating rootlet emanating from the anterior basal body that forms the underlying support for the area of food ingestion [22], [25]. In our study, based on the SEM photos (Fig. 4), the *Pseudobodo* sp. KD51 cells were about 4–5 µm long with an anterior collar around the anterior part of the cell in unstressed feeding cells. Some literatures had reported that the feeding habits of multicellular predators are complexed. First, the prey is drawn to the flagellate by a feeding current that is induced by the beat of one flagellum. It is then captured between the flagella and brought into contact with a sensitive area of the cell surface. A phagocytotic vesicle is formed at this morphologically inconspicuous spot and the prey is ingested. During the digestive process, the food vacuole is transported towards the posterior end of the protist and the undigested remains are subsequently expelled by exocytosis [43]. Hence, in our study, the swimming flagella of *Pseudobodo* sp. KD51 may play important part in the process of alga-predation.

We were sure that the pathogen was the bicosoecid *Pseudobodo* sp. KD51 based on the morphological and molecular features. Since the bicosoecid *Pseudobodo* was discovered when we screened the predatory microorganisms but there was no literature directly describing the relationship between protists and alga, we investigated its predatory ability and predatory stability under different abiotic factors in order to understand its character. From the results of the impact and predatory activity of lysate containing *Pseudobodo* sp. KD51 on *C.vulgaris* cultures, we noted that at a low concentration, it took about 10 day for the protist to crack the host algal cells. Low nutrient levels and low protist concentrations may have been the reasons for the long period of infection. We speculated that *Pseudobodo* sp.KD51 had to directly kill the host and then draw nutrition from the released materials. Another reason may be that the host, *C.vulgaris*, has a growth cycle that is relatively short [64], [65]. When the mother cell of host divide, about four daughter cells form which could partly offset the decrease of the total numbers. To confirm whether *Pseudobodo* sp. KD51 could directly affect the cell wall of the host, we must have to conduct some enzyme activity experiments, such as cellulase and pectinase. As there were reports about the inhibitory mechanisms through hydrolysing cell wall [47], [66].

The relationship between growth rate and temperature for temperate and tropical species of phytoplankton is well established. Eppley [20] demonstrate that the upper limit for phytoplankton growth rate is exponentially and positively correlated with temperature. Growth rates of herbivorous protists have been reported over the temperature range of 5–30°C. In our study, lysate after incubation at a higher temperature (>40°C) for 30 min did not exhibit predatory activity, indicating that *Pseudobodo* sp. KD51 was heat-sensitive (Fig. 6A). Since it was reported that the optimum growth temperature for *P. tremulans* (ATCC PRA­184) on ATCC medium 1525 was 4.0°C, we deduced that the *Pseudobodo* sp. KD51 in our study was a temperate species. There were few reports concerning the effect of pH on the predatory activity of *Pseudobodo*. In fact, Pedersen [67] divided six marine heterotrophic protists into two groups: pH-tolerant species and pH-non-tolerant species. The tolerant group consisted of *Balanion. comatum*, which experienced a reduction in growth when pH exceeded 9.5, and *Oxyrrhis. marina*, which maintained its maximum growth within the pH limit of the experiment (pH 9.9). The pH-non-tolerant group consisted of three ciliates and one dinoflagellate. In our study *Pseudobodo* sp. KD51 was pH tolerant since it retained its predatory activity after treatment under different pH, except those exceeding 9.5, when a sharp decrease of the activity was discovered (Fig. 6B). With regard to the salinity effects on protists, Moreno described multiple effects of salinity on the photosynthesis of the protist *Euglena gracilis* [68]. However unlike *Euglena gracilis, Pseudobodo* sp. KD51 had no chloroplasts, hence, how salinity affected its growth and predatory activity was unknown and awaits further research. Our study, indicated that *Pseudobodo* sp. KD51 could be salinity tolerant since the predation activity, even under high salinity (60%), did not sharply decrease (Fig. 6C).

Bicosoecids consume bacteria in the oceanic environment and might have a direct effect on deep-sea marine fluxes of nutrients. While more research continues on these organisms, much remains unknown about the bicosoecids and other deep-water nanoflagellates and their relationship with other members of phytoplankton. Bicosoecids are bacteriovores, and apparently play a key role in marine flux regulation. Because studies of deep-sea nanoflagellates are relatively recent, more information is currently being gathered in terms of the effect of bicosoecids on the marine environment. In fact, research concerning the relationship between algae and protists still does not go very far, and further work is needed, for example, what is the predatory process, how abiotic factors affect the protist activity, and what about the possible practical application of protists.

## Supporting Information

Figure S1 ***C. vulgaris*** **was co-cultured with pathogen both in liquid and in agar plate condition.** A. Growth of *C. vulgaris* incubated with pathogen on 0, 5 and 10 days post-infection. B. Plaque formation on the *C. vulgaris* lawn plate (90 mm petri dish). Plaques 2 to 3 mm in diameter were seen.
(TIF)

Figure S2 **Growth of** ***C. vulgaris*** **incubated with pathogen under different volume ratio in 8 days.**
(TIF)
