# Supplementary figures and images for: First Report of Pseudobodo sp, a New Pathogen for a Potential Energy-Producing Algae: Chlorella vulgaris Cultures
Source: PLoS One. 2014 Mar 5;9(3):e89571. doi: 10.1371/journal.pone.0089571 (PMC3943784; doi:10.1371/journal.pone.0089571)

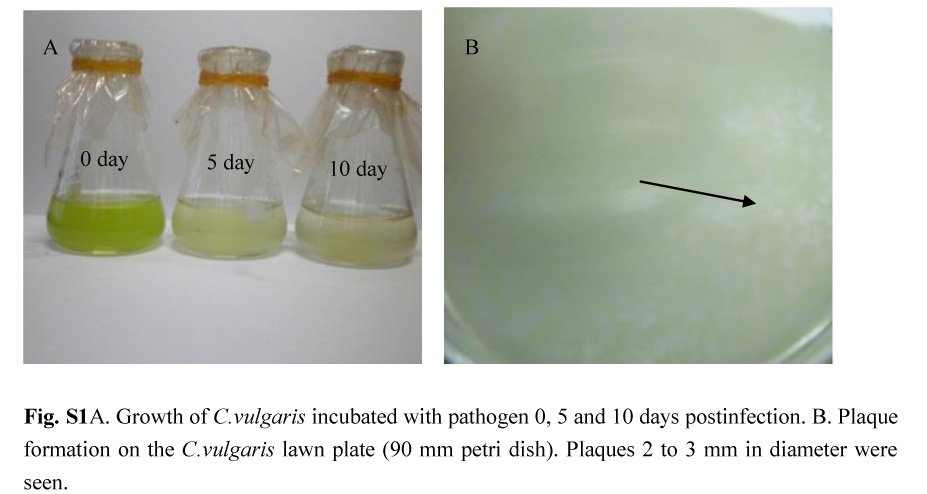

Supplement: Figure S1 — C. vulgaris was co-cultured with pathogen both in liquid and in agar plate condition. A. Growth of C. vulgaris incubated with pathogen on 0, 5 and 10 days post-infection. B. Plaque formation on the C. vulgaris lawn plate (90 mm petri dish). Plaques 2 to 3 mm in diameter were seen. (TIF) [file pone.0089571.s001.tif]

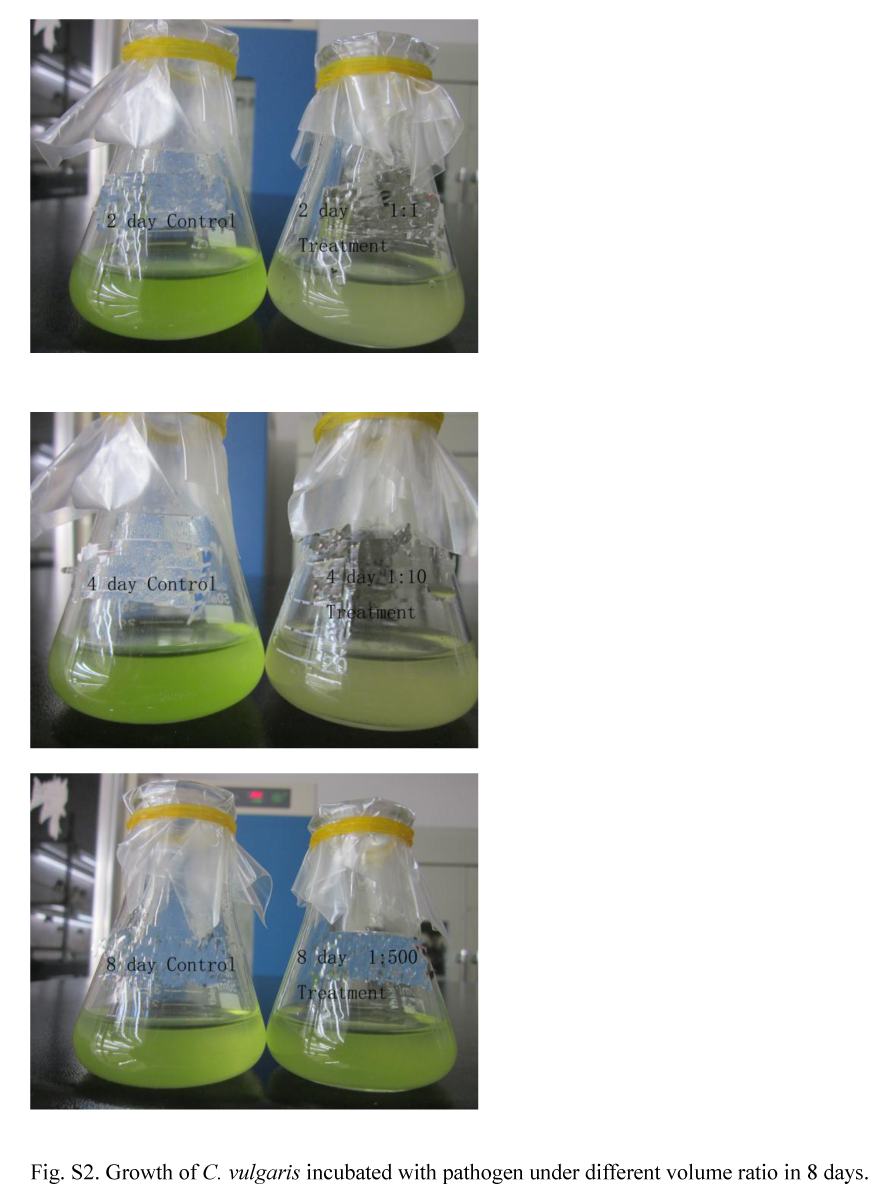

Supplement: Figure S2 — Growth of C. vulgaris incubated with pathogen under different volume ratio in 8 days. (TIF) [file pone.0089571.s002.tif]
